# Kinetics of DNA Repair in *Vicia faba* Meristem Regeneration Following Replication Stress

**DOI:** 10.3390/cells10010088

**Published:** 2021-01-07

**Authors:** Dorota Rybaczek, Marcelina W. Musiałek, Jan Vrána, Beáta Petrovská, Ewa G. Pikus, Jaroslav Doležel

**Affiliations:** 1 Department of Cytophysiology, Institute of Experimental Biology, Faculty of Biology and Environmental Protection, University of Lodz, Pomorska 141/143, 90-236 Lodz, Poland; marcelina.musialek@biol.uni.lodz.pl (M.W.M.); ewa.pikus@biol.uni.lodz.pl (E.G.P.); 2 Centre of Plant Structural and Functional Genomics, Institute of Experimental Botany of the Czech Academy of Sciences, Centre of the Region Haná for Biotechnological and Agricultural Research, Šlechtitelů 31, CZ-77900 Olomouc, Czech Republic; Jan.Vrana@osu.cz (J.V.); beata_petrovska@yahoo.com (B.P.); dolezel@ueb.cas.cz (J.D.); 3 Department of Biodiversity Studies and Bioeducation, Faculty of Biology and Environmental Protection, University of Lodz, Banacha 1/3, 90-237 Lodz, Poland

**Keywords:** caffeine, DNA damage, DNA repair, DNA replication, 5-ethynyl-2′-deoxyuridine, heterochromatin, hydroxyurea, nuclei sorting, premature chromosome condensation, replication stress

## Abstract

The astonishing survival abilities of *Vicia faba*, one the earliest domesticated plants, are associated, among other things, to the highly effective replication stress response system which ensures smooth cell division and proper preservation of genomic information. The most crucial pathway here seems to be the ataxia telangiectasia-mutated kinase (ATM)/ataxia telangiectasia and Rad3-related kinase (ATR)-dependent replication stress response mechanism, also present in humans. In this article, we attempted to take an in-depth look at the dynamics of regeneration from the effects of replication inhibition and cell cycle checkpoint overriding causing premature chromosome condensation (PCC) in terms of DNA damage repair and changes in replication dynamics. We were able to distinguish a unique behavior of replication factors at the very start of the regeneration process in the PCC-induced cells. We extended the experiment and decided to profile the changes in replication on the level of a single replication cluster of heterochromatin (both alone and with regard to its position in the nucleus), including the mathematical profiling of the size, activity and shape. The results obtained during these experiments led us to the conclusion that even “chaotic” events are dealt with in a proper degree of order.

## 1. Introduction

Replication stress is a phenomenon which has serious implications for genome duplication, genome division, and genome stability [1]. Replication stress is generally described as the inhibition or slow-down of DNA replication, which leads to a highly dangerous accumulation of DNA damage in a cell. Eukaryotic cells have developed ataxia telangiectasia-mutated kinase (ATM)- and ataxia telangiectasia and Rad3-related kinase (ATR)-dependent mechanisms that detect and repair this damage [2]; additionally, these pathways prevent the cell from entering the mitosis, preventing the division of incomplete and improperly formed chromosomes [3,4] (Supplementary Figure S1). Failure in resolving cellular replication stress and its consequences usually leads to various diseases, for instance, ciliopathies, cancer, Bloom syndrome, Seckel syndrome, and others, some of which are also linked with events such as mitotic catastrophe (a sequence of events resulting from an inappropriate entry into mitosis and often ending in delayed, mitosis-linked programmed cell death) and/or genome chaos (an extensive chromosome or genome reorganization in response to crisis) [5,6,7,8].

The induction of premature chromosome condensation (PCC) is considered to be one of the first stages of genome chaos in eukaryotic cells. There are several factors that can induce it, however, they all result in the bypass of the S-M and G2-M ATM/ATR-dependent checkpoints of the cell cycle and cause cells with incomplete DNA duplication to start premature mitotic division (Supplementary Figure S1). Cells respond to DNA replication stress by using three partially distinct, but overlapping the intra-S-phase checkpoints, including (i) the replication checkpoint or replication-dependent intra-S-phase checkpoint (which is activated in case of inhibition of DNA biosynthesis) and (ii) replication-independent intra-S-phase checkpoint or double-strand break (DSB)-induced intra-S-phase checkpoint (which blocks mitosis initiation in the case of structural DNA damage, e.g., double-strand breaks, DSBs) together with (iii) the replication-dependent S-M checkpoint, are particularly vital for the well-being of the cell as they are responsible for arresting the entry to mitosis in case of incomplete genome replication [9,10,11,12]. PCC induction forces the cell to enter untimely mitosis and to cope with a couple of stressful factors such as (i) the continuation of unfinished replication, (ii) DNA repair and (iii) DNA condensation simultaneously. Although processing all of these events at the same time seems impossible (and, indeed, for most of the organisms it usually leads to programmed cell death, PCD), our findings suggest the *V. faba* cells might be able to regenerate if given enough time (and water).

Mitotic cell death is a pathway found across the entire eukaryotic domain. A loss of ATR signaling causes severe problems as this kinase is a key signaling protein in the replication stress response system. Deficiencies of Chk1 or ATR contribute to cancer predispositions [13,14]. Therefore, *V. faba*’s survival abilities related to the efficiency of ATM/ATR-related responses may indicate the “weak links” in the replication stress response pathways in humans, whose analogous response mechanisms are highly similar.

In our experiments, we used the combined effects of 2.5 mM hydroxyurea (HU) and 5 mM caffeine (CF) to activate the ATR-dependent checkpoint and induce PCC. Hydroxyurea causes replication stress by reducing the pool of deoxyribonucleoside triphosphates (dNTPs) in the cell nucleus [15] which stalls replication forks. This event poses a threat of extensive accumulation of DNA damage sites [16,17,18,19]. ATR and ATM kinases both play a role in the response to the replication stress by activating the G2 → M cell cycle checkpoint (G2-M or principal control point II (PCP II)) and preventing the cell from entering mitosis with DNA not fully replicated [20]. Caffeine is a widely accessible agent known for its inhibition of ATR and ATM kinases [11,17,21]. Introducing CF to the mixture represses the activation of the G2-M checkpoint and allows the cell to start mitotic division even with unreplicated DNA (Supplementary Figure S1) [17]. We have assessed the levels of DNA damage at the end of the induction period and performed an in-depth analysis of the events occurring during the 12-h long regeneration. The heterochromatin (HC) regions were of the highest importance to us because they enter PCC unreplicated due to the replication arrest caused by HU [20,21,22]. The HC regions are usually defined as geneless, transcriptionally silent parts of the genome that remain tightly packed through most of the cell cycle. HC domains are usually formed of clutches that are denser, larger and not as mobile as euchromatin (EC) [23]. However, they play an important role in chromosome segregation as well as in genome stability [24]. Constitutive heterochromatin is the most transcriptionally “silent” and remains condensed through almost the entire cell cycle, while facultative heterochromatin is composed of the DNA regions of actively regulated gene silencing [25].

After PCC induction the cells enter mitosis with the heterochromatin areas mostly unreplicated. Our observations were to demonstrate that analyzing the chromatin structure can help understanding of the response of *V. faba* root meristem cells to replication stress, as well as the fact that the heterochromatin regions seem to be an essential factor in determining cell survival.

Due to the fact that our research involves events such as DNA replication and mitosis, we decided to investigate actively proliferating root meristem cells of *V. faba*. They have the shortest cell cycle span of all the cells that make up the whole organism of the *V. faba* plant and the majority of them are actively proliferating.

In the present study, we showed that root meristem cells of *V. faba* subjected to chemically-induced replication stress caused by HU and co-treatment with CF show high resistance (and therefore survival rates) to harsh environmental conditions. In our opinion, this ability results from highly efficient stress-response mechanisms during DNA replication. *V. faba* is widely cultivated in dry and hot areas such as Egypt or Saudi Arabia due to its environmental resistance [26]. The impact of salinity does delay the N-fixation of *V. faba* but it cannot affect the rhizobial growth and does not prevent nodulation or N supply to shoots [27]. Moreover, the potential nutritional and functional properties of *Vicia’s* protein isolates make it a desired target for selective growth [28]. Other grain legumes also exhibit similar features [29,30,31]. We analyzed *V. faba* as a model organism as the pathways affected by replication stress are corresponding through all Eukaryotes.

## 2. Materials and Methods

### 2.1. Growth Conditions of Plant Material, Induction of Replication Stress, PCC and Regeneration Period

The seeds of *Vicia faba* var. *minor* (Center for Seed Production, Sobiejuchy, Poland) were dark-germinated at room temperature, until the seedlings were approximately 3 cm long. They were then divided into three experimental series: (i) negative control (Ctrl), incubated for 24 h in distilled water and then transferred to fresh water for another 8 h; (ii) positive control (HU), the seedlings were incubated for 24 h in a 2.5 mM solution of hydroxyurea (HU) and then transferred to a new solution of 2.5 mM HU for another 8 h (HU, an inhibitor of ribonucleotide reductase (RNR), reduces the pool of dNTPs and affects replication fork progression [32]) and (iii) induction of premature chromosome condensation (PCC), the cells were incubated for 24 h in 2.5 mM HU and then transferred to a new mixture of 2.5 mM HU and 5 mM caffeine (CF, methyloxantine disturbing the molecular organization of the S-M checkpoint, which is known as a broad-spectrum nonspecific inhibitor of ATR/ATM [4,11,17,21]). Total times of all incubations were equal to 32 h.

At the end of the incubation period, the DNA damage levels were measured, and all the seedlings were transferred to the fresh distilled water. The levels of DNA damage and the progression of replication were measured after 0.5, 1, 2, 5, 8 and 12 h of water treatment, for each of the experimental series as shown in Supplementary Figure S2.

### 2.2. Comet Assay

The alkaline and neutral comet assays were performed according to the modified protocols of [33,34], respectively. SSBs and DSBs were assessed with alkaline and neutral single-cell microgel electrophoresis (comet assay), respectively, according to the method described by Potocki et al. [35] and Rybaczek et al. [17].

Two preparations were performed for the DNA damage tests (at 32 h of incubation time) and two preparations were performed after each time of repair incubation (for alkaline and neutral electrophoresis).

The protoplasts were isolated from the top meristems of *V. faba* roots, cut off at a length of about 1–2 mm from the apex and macerated. The macerate was next filtered through the gauze and centrifuged for 5 min at 4 °C and 8000 rcf. The isolated precipitate containing protoplasts was suspended in 37% LMP agarose solution (30 µL each sample) and applied on the degreased slides coated with NMP agarose. Cooled slides were placed in the lysis solution for 12 to 24 h and rinsed and incubated for 30 min with an appropriate electrophoretic buffer. The conditions for the electrophoresis were as follows: (i) alkaline 200 mA/15 V/30 min and (ii) neutral 250 mA/40 V/30 min. At the end of the electrophoresis, the slides were washed in the neutralizing buffer twice for 30 min.

Finally, the slides were stained with 0.25 µM YOYO-1 (Invitrogen Corporation, Grand Island, NY, USA) in 2.5% DMSO and 0.5% sucrose (CAS registry number 57–50-1) mounted with a coverslip and digital comet images were immediately captured with an Olympus BX61 fluorescence microscope equipped with a DP72 CCD camera and Olympus CellF software. The CCD capture conditions were: exposure time 100 ms, magnification 400. Images were saved as TIFF files and approximately 150 cells per sample were captured. Results were measured with OpenComet plugin (Laboratory Imaging, Prague, Czech Republic) for ImageJ [36] (NIH and LOCI, University of Wisconsin, Madison, WI, USA).

### 2.3. Flow Cytometric Analysis

Isolation of cell nuclei was performed according to the method described by Wear et al. [37] using: (1) MgSO_4_ buffer: 10 mM MgSO_4_ ·7 H_2_O, 50 mM KCl, 5 mM HEPES (pH 8.0); (2) extraction buffer A: MgSO_4_ buffer with 1% (w/v) polyvinylpyrrolidone (PVP-40), 6.5 mM dithiothreitol, 0.25% (v/v) Triton X-100; (3) extraction buffer B: MgSO_4_ buffer with 6.5 mM dithiothreitol, 0.25% (v/v) Triton X-100, 1.25 µg/mL RNase (DNase-free). Prior to flow cytometric analyses, samples were filtered through a 25 µm pore size nylon mesh filter and stained with 4′,6-diamidino-2-phenylindole (DAPI) to a final concentration of 2 µg/mL according to the method described by Vrána et al. [38].

Actively growing roots of *V. faba* were incubated in tap water containing 10 µM EdU (Click-iT EdU Alexa Fluor 488 kit; Thermo Fisher Scientific, Waltham, MA, USA) for 3 h at room temperature. Suspensions of intact nuclei were prepared according to Doležel et al. [39], with minor modifications. Briefly, roots were cut approximately 0.5 cm from apex and incubated in 2% (v/v) formaldehyde solution for 30 min at 5 °C. The roots were then washed three times in Tris buffer for 5 min at 5 °C. Root apices were excised and chopped with a razor blade in 1 mL LB01 buffer on a Petri dish. The crude homogenate was filtered through a 50-µm pore size mesh. The suspension of nuclei was then centrifuged at 400× *g* for 10 min at 4 °C. The supernatant was discarded and the pellet resuspended in 500 µL Click-iT reaction cocktail (prepared according manufacturer’s instructions) and incubated for 30 min in the dark at 25 °C. Next, the nuclei were pelleted again and the pellet was resuspended in 500 µL LB01 buffer containing DAPI (0.2 µg/mL final concentration).

All flow cytometric experiments were carried out on a FACSAria SORP flow cytometer and sorter (BD Biosciences, San Jose, CA, USA). DAPI fluorescence (DNA content) was excited by UV laser (355 nm, 100 mW) and collected through a 450/50 band-pass emission filter. Alexa Fluor 488 fluorescence (EdU) was analyzed using blue laser excitation (488 nm, 100 mW) with a 530/30 band-pass emission filter. Data were analyzed on dual parameter plots of Click-iT^®^ EdU Alexa Fluor^®^ 488 and DAPI. Cell cycle analysis was performed with a LSRFortessa (Becton Dickinson, Franklin Lakes, NJ, USA) using FACSDiva software (Becton Dickinson, Franklin Lakes, NJ, USA). Data of at least 5000 nuclei were collected in each sample. For sorting, regions corresponding to different cell cycle phases were drawn. Nuclei were sorted onto Superfrost microscopic slides for further analysis. Cell cycle phases were determined using ModFit LT version 3.2 software (Verity Software House Inc., Topsham, ME, USA). Observations were performed by an inverted motorized microscope Olympus IX81 equipped with a Fluoview FV1000 confocal system (Olympus, Tokyo, Japan) and FV10-ASW software, and an Axio Imager.Z2 microscope (Zeiss, Jena, Germany) equipped with confocal Andor DSD2 System and iQ3.6 software (Andor, Belfast, United Kingdom of Great Britain).

### 2.4. Heterochromatin Clusters Analysis

The fluorescence of the nuclei was assessed using ImageJ. First, the images of negative, reference control (the nuclei not displaying the replication fluorescence) were examined. Their mean gray value (the value of lightness, measured from 0—black to 255—white) was assessed and used as the benchmark to distinguish replicating areas. Unedited images (AlexaFluor 488, Thermo Fisher Scientific, Waltham, MA, USA) from the experimental series were transformed into grayscale and at this point the maximum and mean gray values were measured (excluding the background). Next, the images were thresholded and the regions displaying a fluorescence greater than 56 ± 10 (mean gray value for the negative fluorescence) were considered as actively replicating. Upon this procedure, the binary images of the nuclei were obtained. These images were used to measure (i) the number of replication clusters per nucleus, (ii) the average size of replication clusters, (iii) cluster circularity and (iv) cluster solidity. Additionally, the measurements of the total area of nuclei were performed by means of the DAPI-captured images.

### 2.5. Statistical Analysis and Image Processing

The statistical analysis of the results was performed using Statistica 13.3 PL software (StatSoft INC, Tulsa, OK, USA). The probability of *p* < 0.05 was considered as statistically significant. The differences between groups were calculated with the use of one-way ANOVA followed by Fisher’s LSD test. Moreover, the T-test was occasionally conducted for further analysis. All the Figures were prepared in Adobe Photoshop CS6 and Adobe Illustrator CS6 (Adobe Inc., San Jose, CA, USA).

## 3. Results

**Figure 1:**
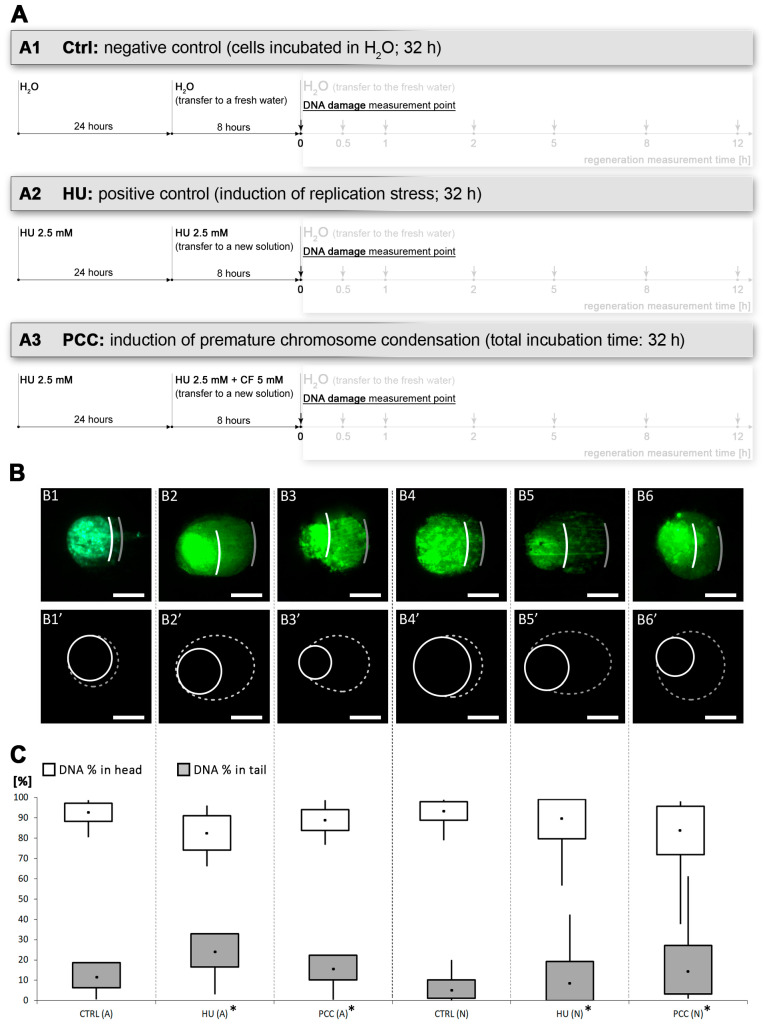
The levels of DNA damage induced by the combined effects of hydroxyurea (HU) and caffeine. (**A**) The diagram of the experimental series, including negative and positive control (**A1** and **A2,** respectively) and premature chromosome condensation (PCC) induction (**A3**). The total incubation time for all subjects was 32 h. (**B**) Alkaline (**B1**–**B3**) and neutral (**B4**–**B6**) comet assay comparison of DNA damage at the end of 32-h incubation time. The cells were stained with YOYO-1. The images presented are representatives from a population of over 100 cells analyzed from each series. Comets were analyzed via ImageJ software with the OpenComet plugin. The photos and drawings are aligned, respectively, to (**C**) the results of the calculation of the percent of DNA in the comet’s tail and head. The statistical significance of the differences was set at * *p* < 0.05 (ANOVA followed by Fisher’s LSD test). The scale bars are equal to 10 µm.

After the induction of replication stress and PCC (as shown on Figure 1A), we observed an accumulation of SSB and DSB sites in cells when compared to the control group (assessed by the alkaline and neutral comet assay measurements, respectively, and ANOVA followed by Fisher’s LSD test at *p* < 0.05; compare with Figure 1B,C). The findings were consistent with the previous experiments on this type of DNA damage [4] and proved the viability of our experimental system. After the assessment of DNA damage levels after 32 h of incubation, we started to measure the efficiency of the regeneration process.

### 3.1. Replication Restarts and Bursts just after the Release from the Replication Block or PCC

The flow cytometry measuring the fluorescence was performed in addition to the comet assay tests just after the release from the 32-h incubation time for the series. A total of 0.5 h before the incubation ended, the samples were transferred to a 100 µM EdU solution. Thus, we could observe the effects of 30-min long replication outcomes at the very end of replication stress and the start of PCC.

**Figure 2:**
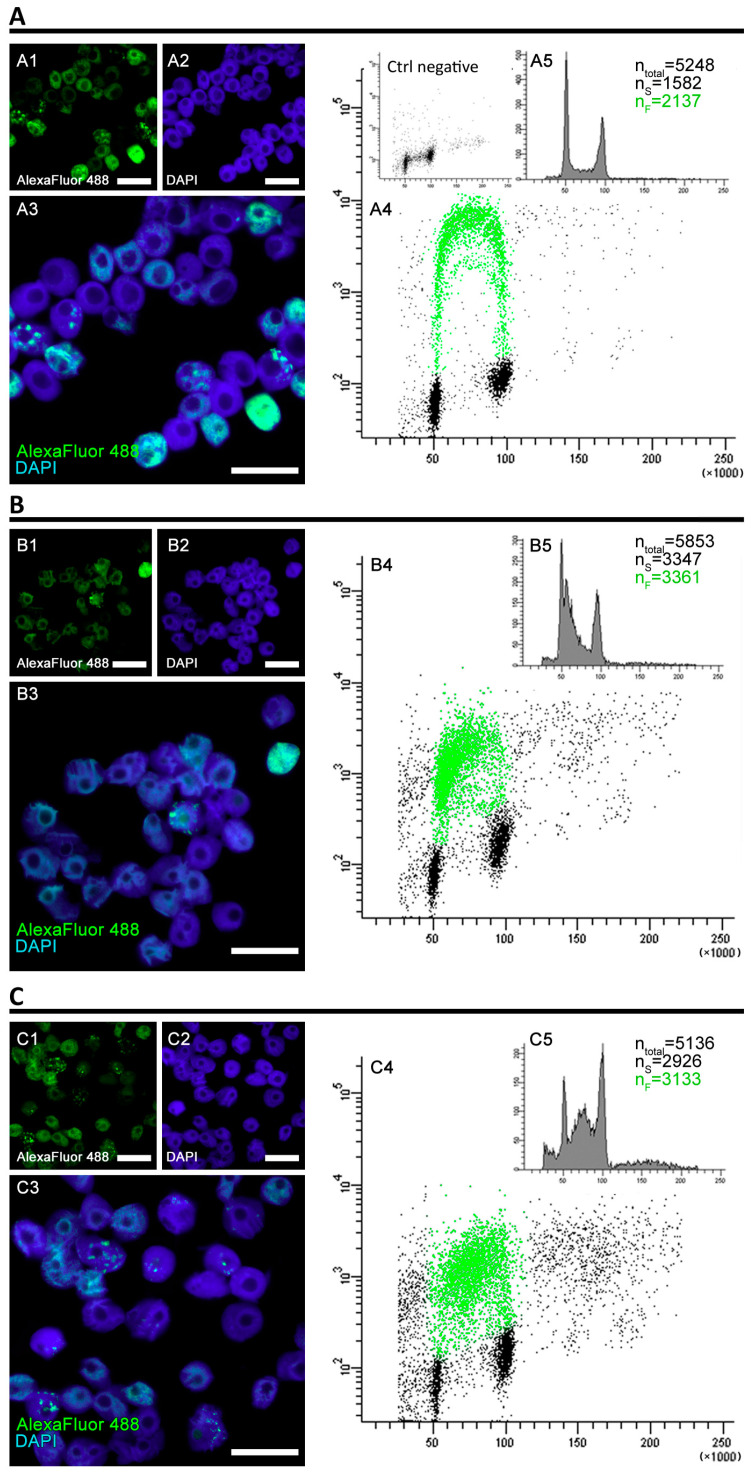
The analysis of fluorescence levels and distribution of replicating clusters after 32 h of cell incubation. The cells were incubated in 100 µM EdU water solution for 0.5 h before the end of the 32-h experimental period and at the end of the incubation, the flow cytometry was performed. (**A**) Positive control, (**B**) negative control (induction of replicative stress), (**C**) PCC induction. The cells were stained with 4′,6-diamidino-2-phenylindole (DAPI) and antibodies conjugated with AlexaFluor 488. The binary images (**A4**,**B4**,**C4**) were prepared based on the thresholded originals. All the photographs are of the cells sorted by the flow cytometer’s sorting unit and include only the S-phase fraction. The scale bars are equal to 10 µm.

Although the DNA is vastly damaged, the HU and PCC cells are quickly capable of restarting replication, though the profiles of cell populations with regard to the cell cycle differ from the control. The fluorescence measured by the flow cytometer is significantly lower for the HU and PCC series (see Figure 2(A4,B4,C4)). We observed that despite former inhibition, a large fraction of the cells restarted replication upon being released from the replication block (as shown in Figure 2(B5)—the number of cells showing fluorescence is 57.4%) and immediately entered the S-phase (as expected). A similar event occurred for the PCC cells (as shown in Figure 2(C5)—61.01% of cells were fluorescent). The numbers of S-phase cells (the n_S_ values) are very similar to the fluorescent cells (the n_F_ values) for HU and PCC, however, they differ from the control (compare Figure 2(A5)).

### 3.2. The Profiles of Heterochromatin (HC) Replication Clusters Are Altered in the PCC-Induced Cells

**Figure 3:**
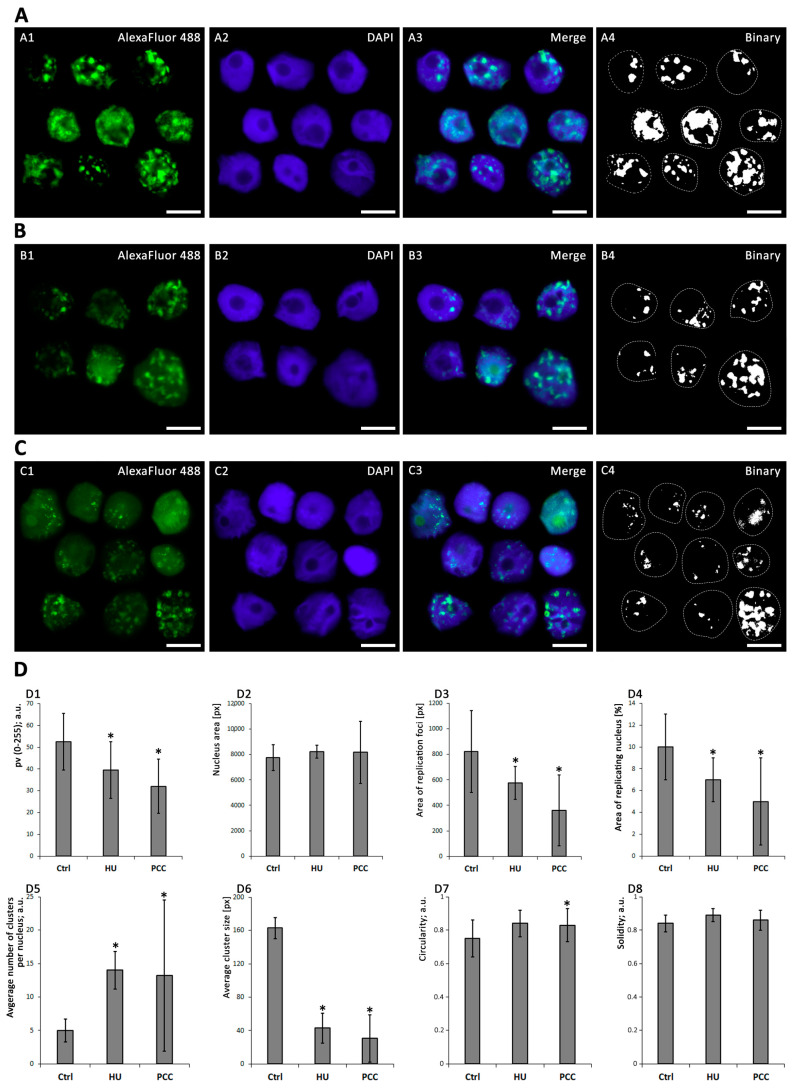
The different development of heterochromatin replication clusters after 32-h incubation. (**A**) Negative control, (**B**) induction of replicative stress by HU, (**C**) PCC induction. The charts (**D**) depict the visual parameters of clusters, measured using ImageJ and analyzed with Statistica 13.3 PL (n = 30 for each series). The nucleus area (**D2**), area of replication loci (**D3**) and average cluster size (**D6**) are given in pixels (px). The following pictures have been selected and put together as representatives of different cluster appearances (due to chemical treatment). The binary images were obtained from the thresholded images. Statistically significant results are marked with * (ANOVA and Fisher’s LSD test, at the significance level of *p* < 0.05). The scale bars are equal to 10 µm.

The sample cells obtained from flow cytometer’s sorting unit (only S-phase) cells were further analyzed using microscope image analysis. As the PCC cells enter premature mitosis with mostly heterochromatin regions unreplicated, we focused on the constitution of the heterochromatin clusters (Figure 3). From the images obtained, only the cells that expressed the replication of the heterochromatin regions were selected and analyzed. We easily distinguished the different, new, appearance of the clusters in PCC samples (compare Figure 2(A3,C3)). Apart from the “normal” and regular clusters, one can observe a significant fraction of cells with a very small and “point-like” replication foci. These were observed only during the first hour after PCC cells were released into the water.

The mean gray value of the clusters (see Figure 3(D1)) describes the relative intensity of replication as the lightness of the pixels on the scale from 0 (black) to 255 (white). We observed significantly lower values for the HU and PCC series, compared to the control (ANOVA and Fisher’s tests, *p* < 0.05). Nucleus area (Figure 3(D2)) was measured from DAPI images and is specified as a pixel area by ImageJ software. The average percent of replicating nuclei (Figure 3(D4)) was assessed as the total area of replication (Figure 3(D3)) divided by the nucleus area. We observed that there is a significant decrease in the area % of replicating nuclei. Additionally, a closer look at single clusters revealed that for HU and (especially) PCC cells, the average number of clusters per nucleus is greatly increased (Figure 3(D5)) while the average cluster size decreases significantly (Figure 3(D6)).

### 3.3. The Amount of DNA Damage Sites Are Temporarily Increased during the Repair Process, the Regeneration from PCC Induction Is Slower than the Regeneration from the Replication Stress

**Figure 4:**
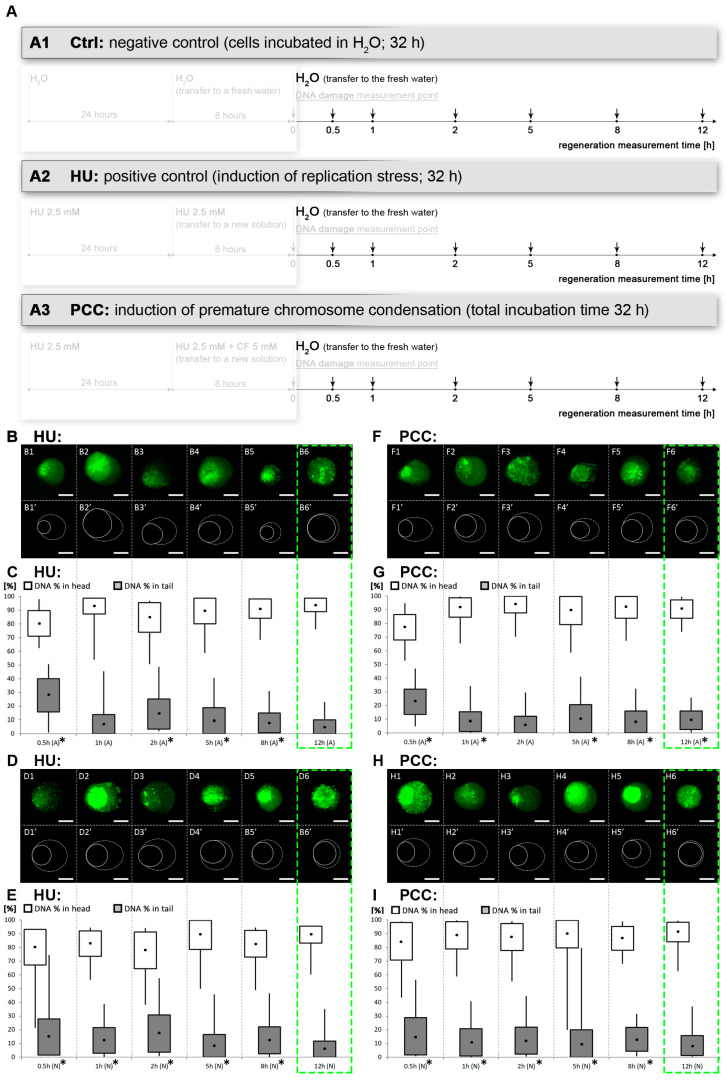
The dynamics of DNA damage repair. (**A**) The diagram depicting the regeneration period and measurement times. After the 32-h incubation, all the cells were released into distilled water, the DNA damage levels were assessed and used as the reference for further measurements. The arrows pointing down show the times of the measurements. (**B**,**C**) Alkaline comet assay for the positive control (HU), (**D**,**E**) neutral comet assay for the HU. (**F**,**G**) Alkaline comet assay and (**H**,**I**) neutral comet assay for the PCC. The drawings marked as **B1′**–**B6′**, **D1′**–**D6′**, **F1′**–**F6′** and **H1′**–**H6′** are based on the microscope images above; the solid line depicts the comet’s head, the dashed line depicts the comet’s tail. The green, dashed frame marks the estimated regeneration time. Both the HU and PCC series were statistically checked for differences against the negative control. The statistical significance was assessed for HU and PCC in comparison to the control at * *p* < 0.05 (ANOVA and Fisher’s LSD test). The scale bars are equal to 10 µm.

The assessment of the DNA damage repair effectiveness was conducted using the alkaline and neutral comet assay. The samples were subjected to the assays during the regeneration period according to the diagram shown in Figure 4A. We observed that levels of DNA damage are highly increased during the first 30 min after water release—the amount of DNA in the tails was the highest of all for the alkaline assay (for both HU and PCC), vastly differing from the control (see Figure 4C,G).

The percentage of DNA in the tail remains significantly different from the control for the majority of the time (up to 8 h after being released into water). We observed that these values came back as similar to the control after 12 h—the % of DNA in the tail was not significantly different from the control samples (ANOVA and Fisher’s test with *p* < 0.05 as a significance level). This conclusion is true for the HU samples for both alkaline and neutral variations (see Figure 4C,E); however, after 12 h in water, the alkaline comet assay for PCC still demonstrated a significant difference (Figure 4F). The neutral assay for PCC showed a “normal” tail, indifferent from the control which points to the conclusion that there is still an excessive amount of SSBs in PCC after 12 h of regeneration. Based on the evidence, we conclude that the regeneration after PCC induction must occur more slowly than from only the replication inhibition (the HU samples).

### 3.4. The Starting Point of the Regeneration Is Vital for the Whole Regeneration Process

**Figure 5:**
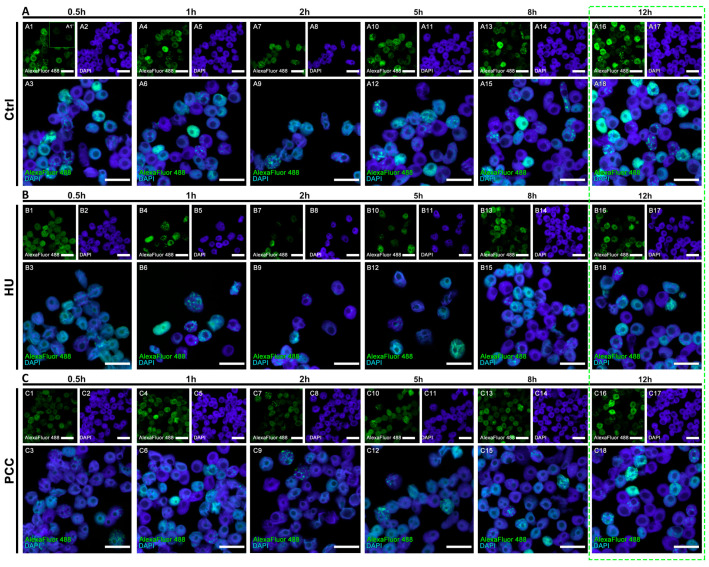
Populational images of the cells during the regeneration period. All of the images contain the cells sorted by the cytometer’s sorting unit (the S-phase fraction only). (**A**) Negative control, (**A1′**) shows the fluorescence reference cells that did not display the fluorescence with AlexaFluor 488. (**B**) HU and (**C**) PCC cell populations. The green dashed frame marks the estimated regeneration time. The scale bars are equal to 10 µm.

PCC-induced cells demonstrated an unusual look in the actively replicating heterochromatin clusters. In control cells, the heterochromatin regions are replicated in clusters of approx. 153 px (as measured by ImageJ software), while the PCC-type clusters were on average 27-pixels large (compare Figure 3(D6)). Even though the clusters from the HU samples are significantly smaller than the control (approx. 50 px in size), they are still uniform in size as given by the standard deviation of 17.869 (compared to the standard deviation for PCC—28.852). These data led us to the conclusion that the PCC clusters are more diversified. There are cells demonstrating very tiny, “point-like” replication clusters in PCC samples (compare Figure 3(C3)). They are, however, unique only to the first hour of the regeneration period and cannot be observed later (see Figure 5C).

**Figure 6:**
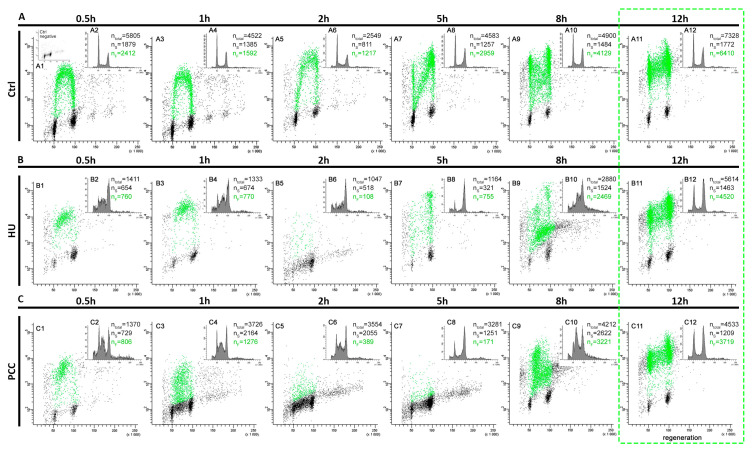
The flow cytometry analysis of cells displaying the fluorescence during the regeneration period. A total of 0.5 h prior to the measurement, the cells were incubated in 100 μm EdU solution. The positive fluorescence (the cells marked with green) signal means that the cells are actively replicating. (**A**) Negative control, (**B**) induction of replication stress, (**C**) induction of PCC. The green dashed frame marks the estimated regeneration time. The small indent in (**A1**), marked as “control negative” is the control 3D histogram of the cells that were not incubated with EdU. n_total_ is the total number of cells measured by the flow cytometer for the period, n_S_ is the number of the S-phase cells and n_F_ is the number of the fluorescent cells at the time.

The flow cytometry analysis of cell fluorescence levels revealed that 12 h are sufficient for the cells to regenerate and restore their replication profile (Figure 6). We discovered, however, that the profiles of PCC and HU vary greatly during the regeneration period. The number of fluorescent cells is usually greater than the number of S-phase cells in control samples (due to the fact that very early and very late replicating cells tend to be marked as G1 or G2 cells by the analyzing software). The replication profiles, however, are easily readable. We observed the deviations from this constant for the HU at the 2nd hour of regeneration and for PCC at the 2nd and 5th hours (see Figure 6(B5,C5,C6)). Here, the number of S-phase cells is much larger than the fluorescent cells and is not related to the sample sizes (n_total_). The fact that there is a large quantity of cells that are described as S-phase, but do not show the fluorescence, may indicate that there is some other mechanism active during this period of time—other than replication and repair.

**Figure 7:**
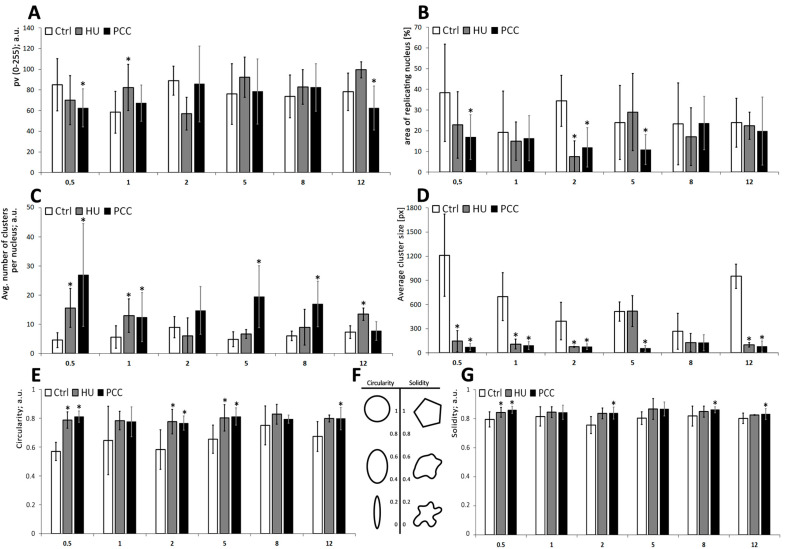
The descriptional parameters of replication clusters, measured during the regeneration period. (**A**) The mean gray value of the nuclei, corresponding to the intensity of the replication. (**B**) The percentage of the area of the nucleus undergoing replication at a given time. (**C**) The average number of replication clusters per nucleus. (**D**) The average size of an individual cluster (measured in pixels). (**E**) Cluster’s circularity—the parameter equals to 1 if the cluster is perfectly round. The lower the circularity, the more elongated the shape is. (**F)** A simple scheme depicting the circularity and solidity. (**G**) Cluster’s solidity—the parameter equals to 1 if the cluster is a regular polygon. The lower the solidity, the less regular the shape is. All of the measurements were conducted with ImageJ, thresholded binary images were used. The statistical significance was assessed for HU and PCC in comparison to the control at * *p* < 0.05 (ANOVA and Fisher’s LSD test).

We decided to analyze the individual replication clusters during the regeneration period as well as the general profiles of DNA replication. The gray value analysis shows that even after 12 h of regeneration time, the average replication intensity is lower in PCC-induced cells, compared to the control (Figure 7A). The percentage of the replicating area of nuclei seems to be fairly similar during most of the periods (with the exception of 0.5 h for PCC, 2 h for PCC and HU and 5 h for PCC, where ANOVA and Fisher’s tests resulted in the decline of the null hypothesis with the *p* value of 0.05). Given that the analysis was done on the cells that were first analyzed and sorted by the flow cytometer, the results for 2 and 5 h may be connected with the results obtained from the 3D histograms (Figure 6(B5,C5,C6)).

We decided to measure two unusual factors that can also describe the constitution of replication clusters. Those are (i) circularity—the degree of “roundness” of the analyzed object (on the scale from zero—elongated shape; to one—perfect circle), and (ii) solidity—the parameter describing the regularity of the object (on the scale from zero—a highly irregular shape with a lot of holes and indents; to one—a perfectly regular shape). A more descriptive diagram is shown in Figure 7F. Here we found that PCC-type heterochromatin clusters are significantly more circular in shape when compared to HU or control samples (see Figure 3(D7,D8)).

The shape descriptors analysis reveals that even though the average number of replication clusters changes back to being the same as the control, their size, circularity and solidity still differ significantly (ANOVA and Fisher’s tests at *p* < 0.05). PCC-type clusters are much smaller and because of that they are also more rounded and regular in shape, compared to the control heterochromatin clusters, which may sometimes “pour together” into one big, highly irregular shape.

## 4. Discussion

### 4.1. Starting Point of Regeneration—DNA Damage Repair, Replication Restart and Cluster Constitution

The fact that the overall DNA damage sites are more common in the HU series may be due to the inhibitory action of HU which depletes the dNTP pools in the cell [15]. Thus, the replication itself is inhibited but also the DNA repair mechanisms are depleted of necessary factors. PCC induction forces the cell to enter premature mitosis by overriding the cell cycle checkpoints [4,17,40,41]. This event leads to the mobilization of the cellular DNA repair mechanisms together with the mechanisms that try to continue the replication despite an untimely division.

A significant difference between the number of fluorescent and S-phase cells (Figure 2) indicates the speed of the early replication events. Water-treated, normal cells start the replication quickly because, at the beginning, the transcriptionally active euchromatin regions are replicated [42,43,44]. These regions are also only a small part of the whole plant genome, so the increase in cell weight is not very visible. Replication, however, progresses quickly and thus the fluorescence levels are high. Significantly lower mean gray values of replication activity for HU and PCC can indicate that (i) there are fewer replication areas in HU and PCC cells or (ii) the intensity of replication itself is lower, which may indicate that it progresses slower than the control. Given the fact that the results of an individual cluster’s analysis show that the average cluster size decreases and the total area of replication in the nucleus decreases as well (despite the increase in the number of replication clusters), we conclude that the former option is true.

Heterochromatin clusters measured at the very start of the regeneration period describe the starting points at which the cells started to freely deal with the replication stress and premature mitoses. As stated before, the activity of replication factors (the intensity of replication) resumed its “normal” levels shortly after the regeneration started. This is in line with the results obtained by flow cytometry at the start of the regeneration period (Figure 2) where we observed that the percentage of actively replicating cells was similar throughout the series. The smaller size of HU and PCC-type clusters shows, however, a slower progress of replications, possibly due to the fact that a lot of the sites are simultaneously subjected to the activity of DNA repair mechanisms.

The increased number of replication clusters in PCC and their significantly lower and more circular appearance may be the effect of two factors. Heterochromatin regions are significantly more compact than euchromatin parts of the genome by default [24,45]. In addition to that, PCC induction forces chromatin to become even more dense (because of the start of the premature mitosis process). Along with the fact that replication factors are restarted within these regions in an attempt to finish the replication, the replication forks may become quickly trapped within the congesting net of DNA and proteins, thus resulting in very small, “point-like” replication signal clusters. This type of cluster was observed only during the first hour after releasing the cells into water from the PCC induction. Additionally, the increased number of replication clusters may indicate the events of dormant origin firing in an attempt to catch up with the delayed replication. We have also observed that some fraction of replicating heterochromatin is relocated to the outer regions of the nucleus—probably because of the activity of homologous recombination (HR) mechanisms that require DSB sites to move outside of the main body of heterochromatin to complete recombinational repair [46]. HR is important for DSBs repair at stalled replication forks in all organisms [47].

### 4.2. Regeneration Period

Given that alkaline comet assay is sensitive to both types of DNA breaks (SSBs and DSBs), compared to the neutral assay (sensitive to DSBs), one can conclude, that during the first 30 min of the regeneration period, there is a significant increase in the amount of SSBs. This phenomena may be caused by (i) the activity of DNA damage repair mechanisms which have to induce local DNA cuts in order to successfully repair the nucleic acids [17,48,49] or (ii) the activity of DNA replication mechanisms trying to come to an end with disturbed replication [43,44]. We suspect that both possibilities play a role here.

PCC-caused DNA damage is removed at a slower pace (compared to HU) despite the higher intensity of the mechanism’s activity. Premature mitosis induction, however, forces the chromatin to become dense, which hinders access to the damage sites for the repair enzymes resulting in an intense, yet slow repair (compare Figure 4). The “point-like” replication clusters may be the effect of such events. Additionally, these are only observed during the first hour of the regeneration period. We suppose that the greatest challenge for the PCC-induced cells is the replication and (or) repair of the prematurely condensed heterochromatin regions at the same time. They may not be transcriptionally active yet are still vital for the whole genome integrity as they greatly contribute to the proper chromosome folding and segregation. This process cannot continue forever though, so we conclude that there must be a “break point” when the repair/replication processes are discarded. It is a well-known fact that in the presence of excessive DNA damage that cannot be repaired, the cell enters the programmed cell death pathway [17]. Our preliminary guess is that (as the flow cytometry results show) this event could occur anywhere from 2 to 5 h after release into the water (Figure 6(B5,B7,C5,C7)). The analysis of replication clusters on a single-cluster level also shows the significant differences in the percentage of the replicating nucleus at the 2 and 5-h measurement times (Figure 7B)—the results are coherent with the results from the flow cytometer (2 h for the HU and 2 and 5 h for the PCC).

**Figure 8:**
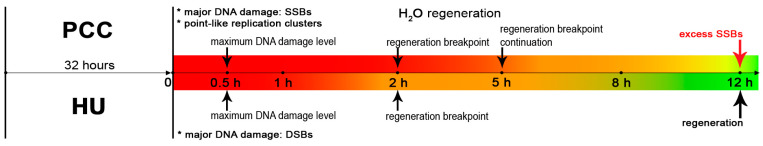
The results of the PCC induction and the major events of the regeneration period. The indicators above the time axis describe the PCC regeneration, the indicators below describe HU regeneration. PCC regeneration occurs slowly compared to HU and is not fully completed after 12 h as previously thought. A total of 0.5 h after the release into the water one can observe the maximum levels of DNA damage, probably caused by the activity of the DNA repair mechanisms. At the 2nd hour, both series achieve the regeneration “breakpoint” characterized by a significantly low number of fluorescent cells (indicating that the major fraction of cells do not replicate at this moment). For the PCC-induced cells this event is prolonged and continues through the 5th hour. After the breakpoint, the regeneration resumes and is completed at the 12th hour for HU. At this time, the PCC series still expressed a significantly higher level of SSBs.

It was shown that the regeneration of meristematic root cells occurs in the following sequence (Figure 8): (1) activation of the repair mechanisms (accompanied by a temporary increase in damage over 0.5 h of regeneration) as well as launching the replication; (2) “breakpoint” in the 2nd hour of regeneration, during which the cell evaluates whether it will be able to repair the damaged DNA and/or replicate the DNA or not, and finally (3) continuation of regeneration in those cells that will survive the “breakpoint”.

## 5. Conclusions

Our studies were carried out on the model of intensively dividing *V. faba* root meristem cells. The main aim of this study was to describe the kinetics of DNA repair during meristem regeneration subjected to: (i) replicative stress induced by HU, or (ii) PCC induced under permanent replicative stress conditions. The results showed high survivability of *V. faba* cells. Other gain legumes also exhibit similar features (i.e., they are tolerant to the salinized soil [27,29,30,31], high temperatures and drought [26]). We chose to investigate *V. faba* among many others because it makes a perfect model organism. The second purpose of the study was to investigate the chromatin structure, with particular emphasis on heterochromatin regions (due to the fact that heterochromatin were the most vulnerable to the occurrence of DNA damage resulting from under-replication). There are few papers which report the analysis of the heterochromatin replication clusters’ shape as mathematical descriptors. The obtained data appear to be sufficient to draw the conclusion that the analysis of heterochromatin clusters, using classic photo analysis programs, turns out to be very helpful and seems to be universal in heterochromatin imaging (not only during replication), but can also be useful in the analysis of proteins related to heterochromatin, e.g., proteins from the HP1 family [50,51].

Taken together, the induction of PCC provides plenty of problems for the cells which have to deal with unreplicated DNA, DNA damages and premature mitotic division at the same time. Even though the damaging effects are quite effectively removed within 12 h, the cells subjected to PCC induction tend to recover slower than the cells subjected only to replication stress. Both processes, however, share some similar and distinguishable events that are timed to ensure the best possible outcome.

Due to the fact that the pathways responsible for the regeneration abilities of *V. faba* are pretty much conserved throughout the eukaryotic domain, a similar response may be the background of various ATR-related diseases in humans. Many of the commonly used chemotherapeutic drugs induce replication stress in cancer cells, forcing them to enter PCD because of the mitotic catastrophe. Further studies on the *V. faba*’s efficiency in executing an ATR-dependent stress response pathway, even upon its partial failure, may bring some new ideas to the treatment of various diseases in humans.

## Supplementary Materials

The following are available online at https://www.mdpi.com/2073-4409/10/1/88/s1, Supplementary Figure S1: A model for the hydroxyurea-induced checkpoint activation and caffeine-induced checkpoint omitting. The mechanisms connected with the intra-S-phase checkpoints are set in motion under the condition of replication stress under the influence of hydroxyurea (HU, an inhibitor of ribonucleotide reductase). HU synchronizes the cells in the S-phase of the cell cycle and—in a long-term exposure—at the S/G2 boundary, which results in the phosphorylation of: (i) Chk1 kinase, (ii) Chk2 kinase, and (iii) S139 of H2AX histone (H2AXS139ph, a marker of double strand breaks (DSBs)) by superior kinases: ATR and ATM (black arrows). Chk1 is activated by the kinases related to phosphoinositide 3-kinases (PIKKs): ataxia telangiectasia and Rad3-related kinase (ATR) and—in a supportive way (black dashed arrows)—ataxia telangiectasia-mutated kinase (ATM). Chk2 and H2AXS139ph are activated by ATM kinase and—in a supportive way—ATR. The activated Chk1 and Chk2 can inhibit entry into mitosis (M). Caffeine (CF, a methylxantine) overrides the HU-induced cell cycle arrest and initiates premature chromosome condensation (PCC) or mitotic catastrophe (red pathway). Based on [4], modified. Supplementary Figure S2: The diagram of the flow cytometry analysis. 0.5 h before the end of the incubation time for all of the series (depicted as “0” on the time axis), the cells were transferred to the 100 µM EdU solution in fresh, distilled water. After 0.5 h incubation the flow cytometry test was performed, the levels of fluorescence were measured and the cells were sorted based on the phase of the cell cycle. The regeneration samples were treated in the same way—they were subjected to a 0.5 h incubation in 100 µM EdU before the measurement. The green arrows pointing up mark the start of EdU incubation, the black arrows pointing down mark the flow cytometry analysis.

## List of Abbreviations

ATM	ataxia telangiectasia-mutated kinase
ATR	ataxia telangiectasia and Rad3-related kinase
CF	caffeine
dNTPs	deoxyribonucleoside triphosphates
DSB	double-strand break
EC	euchromatin
HC	heterochromatin
HR	homologous recombination
HU	hydroxyurea
PCC	premature chromosome condensation
PCD	programmed cell death
PCP II	principal control point II
SSB	single-strand break
