# Supplementary material for: Kinetics of DNA Repair in Vicia faba Meristem Regeneration Following Replication Stress
Source: Cells. 2021 Jan 7;10(1):88. doi: 10.3390/cells10010088 (PMC7825715; doi:10.3390/cells10010088)
Supplement: Supplementary file 1 [file cells-10-00088-s001.pdf]

cells MDPI

Appendix A

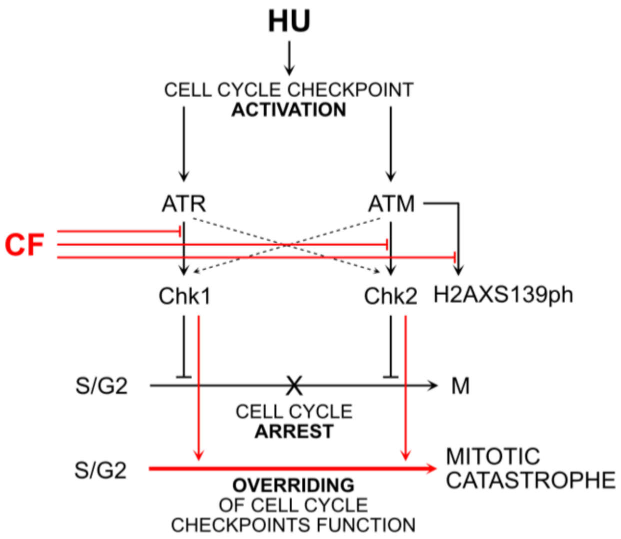

Figure S1: A model for the hydroxyurea-induced checkpoint activation and caffeine-induced checkpoint omitting. The mechanisms connected with the intra-S-phase checkpoints are set in motion under the condition of replication stress under the influence of hydroxyurea (HU, an inhibitor of ribonucleotide reductase). HU synchronizes the cells in the S-phase of the cell cycle and - in a long-term exposure - at the S/G2 boundary, which results in the phosphorylation of: (i) Chk1 kinase, (ii) Chk2 kinase, and (iii) S139 of H2AX histone (H2AXS139ph, a marker of double strand breaks [DSBs]), by superior kinases: ATR and ATM (black arrows). Chk1 is activated by the kinases related to phosphoinositide 3-kinases (PIKKs): ataxia telangiectasia and Rad3-related kinase (ATR) and - in a supportive way (black dashed arrows) - ataxia telangiectasia-mutated kinase (ATM). Chk2 and H2AXS139ph are activated by ATM kinase and - in a supportive way - ATR. The activated Chk1 and Chk2 can inhibit entry into mitosis (M). Caffeine (CF, a methylxantine) overrides the HU-induced cell cycle arrest and initiates premature chromosome condensation (PCC) or mitotic catastrophe (red pathway). Based on [4], modified.

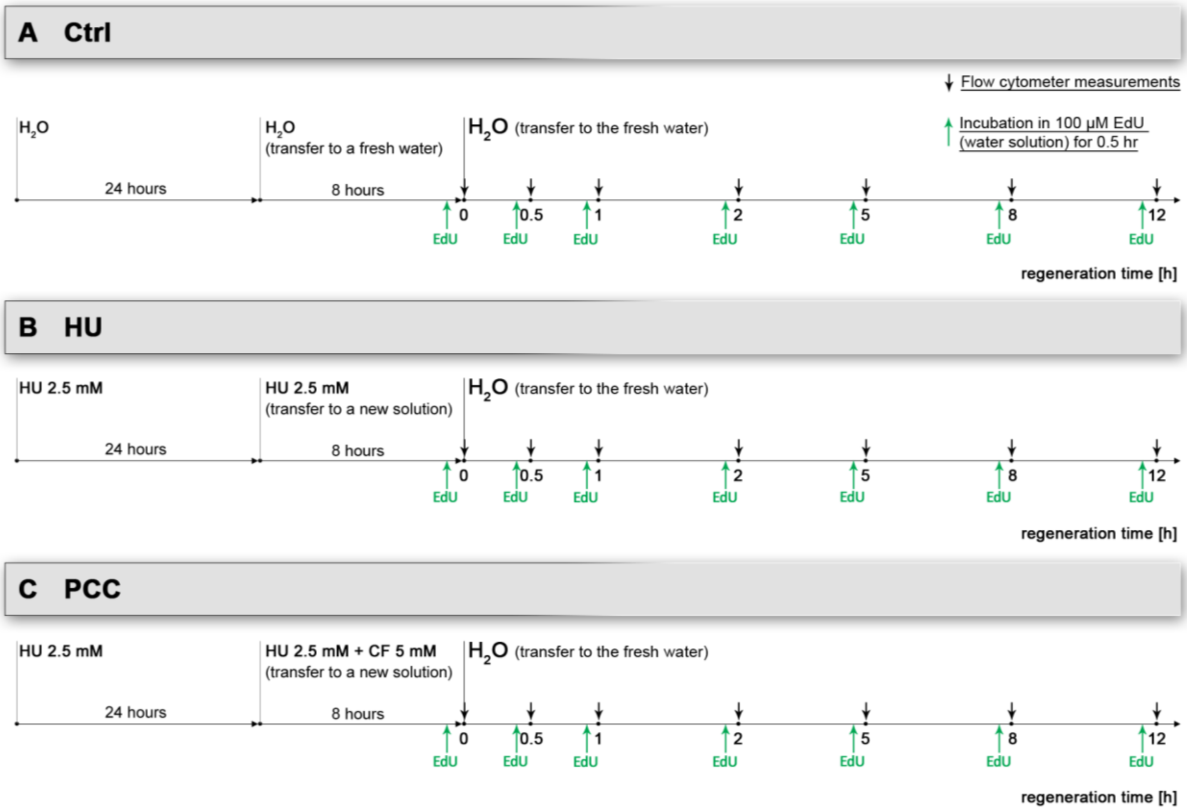

Figure S2: The diagram of the flow cytometry analysis. 0.5 hour before the end of the incubation time for all of the series (depicted as '0' on the time axis), the cells were transferred to the 100 µM EdU solution in fresh, distilled water. After 0.5 hr incubation the flow cytometry test was performed, the levels of fluorescence were measured and the cells were sorted based on the phase of the cell cycle. The regeneration samples were treated in the same way – they were subjected to a 0.5 hr incubation in 100 µM EdU before the measurement. The green arrows pointing up mark the start of EdU incubation, the black arrows pointing down mark the flow cytometry analysis.
